# Human mesenchymal stromal cells-laden crosslinked hyaluronic acid-alginate bioink for 3D bioprinting applications in tissue engineering

**DOI:** 10.1007/s13346-024-01596-9

**Published:** 2024-04-25

**Authors:** Cristina Galocha-León, Cristina Antich, Ana Voltes-Martínez, Juan A. Marchal, Mireia Mallandrich, Lyda Halbaut, Eliana B. Souto, Patricia Gálvez-Martín, Beatriz Clares-Naveros

**Affiliations:** 1 Department of Pharmacy and Pharmaceutical Technology, Faculty of Pharmacy, University of Granada, University Campus of Cartuja, 18071 Granada, Spain; 2 Biopathology and Regenerative Medicine Institute (IBIMER), Centre for Biomedical Research, University of Granada, 18100 Granada, Spain; 3 Biosanitary Institute of Granada (ibs. GRANADA), University Hospital of Granada-University of Granada, 18100 Granada, Spain; 4 Department of Human Anatomy and Embryology, Faculty of Medicine, University of Granada, Granada, 18012 Spain; 5 Excellence Research Unit “Modeling Nature” (MNat), University of Granada, 18016 Granada, Spain; 6 BioFab i3D Lab - Biofabrication and 3D (Bio)printing Singular Laboratory, University of Granada, 18100 Granada, Spain; 7 Department of Pharmacy and Pharmaceutical Technology and Physical Chemistry, Faculty of Pharmacy and Food Sciences, University of Barcelona, 08028 Barcelona, Spain; 8 Institut de Nanociència i Nanotecnologia IN2UB, Universitat de Barcelona, 08028 Barcelona, Spain; 9 Laboratory of Pharmaceutical Technology, Faculty of Pharmacy, University of Porto, 4050-313 Porto, Portugal; 10 R&D Human and Animal Health, Bioibérica S.A.U., 08029 Barcelona, Spain

**Keywords:** Human mesenchymal stromal cells, Bioink, Regenerative medicine, Tissue engineering, Biomaterials

## Abstract

Three-dimensional (3D) bioprinting is considered one of the most advanced tools to build up materials for tissue engineering. The aim of this work was the design, development and characterization of a bioink composed of human mesenchymal stromal cells (hMSC) for extrusion through nozzles to create these 3D structures that might potentially be apply to replace the function of damaged natural tissue. In this study, we focused on the advantages and the wide potential of biocompatible biomaterials, such as hyaluronic acid and alginate for the inclusion of hMSC. The bioink was characterized for its physical (pH, osmolality, degradation, swelling, porosity, surface electrical properties, conductivity, and surface structure), mechanical (rheology and printability) and biological (viability and proliferation) properties. The developed bioink showed high porosity and high swelling capacity, while the degradation rate was dependent on the temperature. The bioink also showed negative electrical surface and appropriate rheological properties required for bioprinting. Moreover, stress-stability studies did not show any sign of physical instability. The developed bioink provided an excellent environment for the promotion of the viability and growth of hMSC cells. Our work reports the first-time study of the effect of storage temperature on the cell viability of bioinks, besides showing that our bioink promoted a high cell viability after being extruded by the bioprinter. These results support the suggestion that the developed hMSC-composed bioink fulfills all the requirements for tissue engineering and can be proposed as a biological tool with potential applications in regenerative medicine and tissue engineering.

**Graphical abstract:**

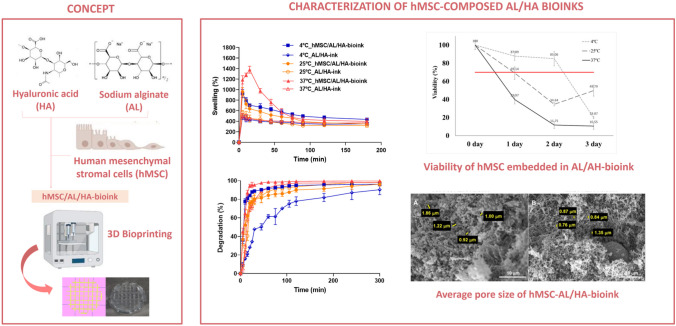

**Supplementary Information:**

The online version contains supplementary material available at 10.1007/s13346-024-01596-9.

## Introduction

Three-dimensional (3D) bioprinting is an innovative technology in biomedical engineering for the biofabrication of 3D structures intended for regenerative medicine and tissue engineering applications. This technique is promoting the progress of tissue engineering (TE) because it provides biomimetic structures by controlled 3D deposition of living cells supported by biopolymers in a spatial location [1, 2]. Bioinks are the materials used to encapsulate cells during the bioprinting of tissues. They provide structure for the bioprinted tissue, and support and nutrients for the cells, providing an optimal environment to promote the survival and maintenance of cells [3, 4].

The inkjet-based bioprinting (i.e., droplet bioprinting), laser-assisted bioprinting (LAB) and extrusion-assisted bioprinting are the most widely used 3D bioprinting technologies. To date, extrusion-assisted bioprinting has been the most commonly type of 3D bioprinting in TE applications due to its affordability, versatility and capacity to build complex constructs [5]. 3D bioprinting technologies have created complex tissue structures with precise control. However, they cannot accurately mimic the dynamic nature of tissues. For this purpose, four-dimensional (4D) bioprinting has been developed using stimuli-responsive biomaterials and cell traction forces, allowing for a more realistic simulation of the dynamics observed in natural tissues. However, 4D bioprinting still needs to evolve to ensure safe and predictable post-printing changes to cell behaviour and function [6–8].

In order, to achieve the desired tissue construct, it is essential to understand the properties of the bioink. An optimal bioink should satisfy specific physicochemical and mechanical requirements to be extruded, and also able to support the structural integrity of the resulting construct [9]. Besides, the bioink needs to ensure the maintenance of cell viability before the bioprinting process [10], and provide an optimal environment for cell proliferation, migration, differentiation and adhesion after bioprinting to become a functional construct [11].

Bioinks are often based on hydrogel frameworks. These are 3D networks made of hydrophilic polymers that can absorb a large amount of water or biological fluids, while maintaining their structural network. The hydrogel should depict adequate properties to mimic the natural extracellular matrix (ECM), and the capacity to provide a suitable environment for the living cells allowing the exchange of gases and nutrients [12]. Bioinks may be composed of natural and/or synthetic biomaterials [13]. Natural biomaterials, such as hyaluronic acid (HA), offers a favorable environment for cell growth. The resulting construct possesses suitable properties, such as biocompatibility, biodegradability and non-toxicity [10, 11]. HA is a glycosaminoglycan, playing an active role as a signaling molecule in cell migration and proliferation, and it has also excellent biocompatibility [14]. Moreover, hydrogel scaffolds made of HA have high porosity, which promotes diffusion of nutrients and waste products [3]. As the molecular weights of HA vary greatly, this property significantly influences the physical, physicochemical, structural and degradable properties of the polymer. The biological effects of HA also depend heavily on its molecular weight. HA of high molecular weight contributes to the osmotic balance, allows the tissue hydration, stabilizes the ECM structure and shows immunosuppressive, anti-inflammatory and antiangiogenic effects. By contrast, HA of low molecular weight induces pro-inflammatory, pro-angiogenic and immunostimulatory responses [15]. On the other hand, HA of high molecular weight (> 1000 kDa) often contains longer chains and more intermolecular entanglement providing higher viscosity. In fact, there is a correlation between the branched-chain length and viscosity [16]. Despite these advantages, HA lacks the required mechanical properties to ensure structural integrity. This circumstance may cause some problems in the bioprinting process due to the reduced rigidity of the structures [14]. To overcome these drawbacks, there is a growing trend toward the use of gelling agents for non-covalent cross-linking of this glycosaminoglycan [17]. The HA can be functionalized or mixed with natural gelling agents, such as alginate (AL), promoting the development of more rigid structures suitable for use as bioinks [18–20]. AL is a natural water-soluble linear polysaccharide with excellent crosslinking properties, that is, it has a remarkable ability to form strong and stable connections (cross-links) between its polymer chains, resulting in the formation of a three-dimensional network structure known as a hydrogel. The use of alginate cross-linking in the manufacture of hydrogels for cell encapsulation has been found to be the most beneficial for biomedical applications [21, 22]. AL also shows good biocompatibility, low cytotoxicity, excellent rheological properties, fast gelation kinetics and high swelling ratio. All these properties mimic the structure of the natural ECM [23]. AL is able to form reticular structures by Ca^2+^ ions facilitating its use in bioprinting [24]. CaCl_2_ has been used in previous studies as part of an AL solution for the generation of hydrogels [25], and it has been also used as a base component in the manufacture of polymers by electrospinning [26, 27] with limited cytotoxic rises. Furthermore, AL hydrogels are able to prevent the death of loaded cells from the host immune response, i.e., they act as a barrier or protection, preventing the host’s immune system from detecting and attacking the loaded cells, allowing the cells to survive [28]. They have also shown great applicability as structures for cell immobilization [29]. Lee et al. [19] described in their research the development of bioinks from hyaluronic acid and sodium alginate at different ratios and the bioprinting 3D constructs through extrusion techniques using all the bionks. Antich et al. [14] prepared a similar bioink consisting of 1% (w/v) HA and 2% (w/v) alginate to produce cartilage constructs. Bioink was co-printed with polylactic acid (PLA) and they suspended humane articular chondrocytes. They confirmed that the mixture of HA with AL provides the suitable mechanical properties to be used as cell-carrier biomaterial for construct fabrication by 3D bioprinting. Both works confirm the approach described above about using hyaluronic acid in combination with other polymers to improve the properties of bioinks. Despite significant progress in tissue engineering, challenges remain in optimizing of HA bioinks. The modification of HA is a recent strategy, providing this polysaccharide with new features. HA is commonly modified with UV-curable methacrylate (MA) to render it photopolymerizable. This modification preserves the essential biological characteristics of HA while acquiring controllable cross-linking properties, significantly enhancing cross-linking efficacy and mechanical stability for scaffold bioprinting [30, 31]. A bioink kit based on a methacrylated derivative of hyaluronic acid (PhotoHA™-IRG) has been commercialised by Sigma-Aldrich [32]. In this topic, Hauptstein et al. [33] developed HA-bioinks using thiol-modified hyaluronic acid and its crosslinking with acrylated (PEG-diacryl) and allylated (PEG-diallyl) polyethylene glycol, demonstrating that about 98% of the MSCs were still viable in the constructs after extrusion procedure.

An essential aspect in the design and formulation of bioinks is the choice of the cell type to combine with biomaterials and bioactive molecules to improve an effective application. The use of stem cells in bioprinting offers a great deal of advantages, such as the ability to induce immune tolerance and to expand once incorporated into the target tissue. Specifically, hMSC are good candidates for TE due to their immunomodulatory and multipotency properties, their paracrine effects and homing abilities towards tissue repair [34]. It has been demonstrated that hMSC are able to stimulate immune tolerance in target tissue [4, 35]. They have also in vitro differentiation potential into osteocytes, adipocytes and chondrocytes [36]. Another reason for the selection of hMSC for tissue regeneration is the capability of these cells to induce several bioactive trophic factors that stimulate neighboring parenchymal cells towards repairing injured tissues. Moreover, they are able to migrate to damaged sites and support the regeneration processes [37]. hMSC are used in cartilage repair, regeneration of musculoskeletal tissues, skin, liver and bone, rebuilding of central nervous system and peripheral nervous system, myocardium restoration, reconstruction of corneal, and tracheal reconstruction [38].

Due to the advantages and the wide potential of HA and AL in TE, the main objective of this work was the development and characterization of a hydrogel based on AL and HA as bioink loaded with hMSC for its application in the biofabrication of 3D tissues and organs. The specific goals were: (*i*) the detailed physicochemical characterization of the bioink, including the effect of the temperature of storage on cell viability, (*ii*) the study of its mechanical properties, (*iii*) the study of its biocompatibility, and (*iv*) the assessment of the 3D bioprintability to create constructs that might potentially be applied to replace the function of damaged natural tissue.

## Materials and methods

### Chemicals and reagents

All reagents and chemicals were of analytical grade and purchased from Sigma-Aldrich (Madrid, Spain) unless otherwise stated, and used without prior purification. Hyaluronic acid (HA, 1000 kDa), was kindly gifted by Bioibérica S.A.U. (Barcelona, Spain). Low viscosity sodium alginate (AL, 4-12 cP; CAS number 9005-38-3; molecular weight 216.12) was obtained from Sigma-Aldrich (Madrid, Spain). A Milli-Q^®^ Gradient A10 system apparatus (Millipore Iberica, Madrid, Spain) was used for the filtration of double distilled water prior to its use.

### hMSC isolation and culture

Subcutaneous adipose tissue was collected, upon informed consent, from patients that underwent liposuction procedure. This study was approved by the Ethics Committee of Clinical University Hospital of Málaga (Spain) (number: 02/022010). The isolation and culture protocols of hMSC were performed following the work by López-Ruiz et al. [38]. hMSC were characterized following the established criteria of the International Society for Cellular Therapy (ISCT) [39], and following the protocols described by Martínez-Moreno et al. [40]. Adipose tissue collected from lipoaspiration was minced and treated on a shaker by enzymatic digestion solution of 1 mg/mL of collagenase type IA, at 37 °C for 1 h. After digestion, the enzyme collagenase was removed by a single wash in sterile phosphate-buffered saline (PBS), and washed twice with Dulbecco’s Modified Eagle Medium (DMEM) supplemented with 10% fetal bovine serum (FBS) (Invitrogen Inc., New York, USA). The cell pellet was suspended in DMEM containing 10% FBS and 1% streptomycin/penicillin, and cultured at 37 °C in 5% CO_2_. After 48 h, non-adherent cells were discarded from the removed medium. When 80% level of confluence was reached, cells were released with TrypLE (Invitrogen Inc., New York, USA) and sub-cultured. For all the experiments, hMSC were used between passages 4 and 6.

### Preparation of AL/HA-bioink loaded with hMSC

The formulation of the bioink was carried out in three stages. First, the hydrogel was prepared by dissolving AL and HA in PBS at concentrations of 6% (w/v) and 1.33% (w/v), respectively, obtaining a non-crosslinked AL/HA-based hydrogel (AL/HA-hydrogel) [41]. Previously, both materials were sterilized by short cycle autoclaving. This techniques has been previously shown to be effective in the sterilization of materials such as HA against some types of bacteria without affecting the rheology, physicochemical prop-erties and printability [42] and it has also been used to sterilisation of the AL in others previous works [28, 43, 44]. Second, non-crosslinked AL/HA-hydrogel was combined with 100 mM CaCl_2_ (3:1) to form the semi-crosslinked hydrogel, being the final concentrations 4.5% (w/v) and 1% (w/v) for AL and HA, respectively. The semi-crosslinked hydrogel was mixed homogeneously using a 3 cc Luer-Lock syringe with a connector and stored at 4 °C until use. Briefly, hMSC pellet were suspended in the semi-crosslinked AL/HA-hydrogel at the concentration of 1 × 10^6^ cells/mL resulting the hMSC/AL/HA-based bioink (hMSC/AL/HA-bioink). Then, hMSC/AL/HA-bioink packed in 3 cc syringes was gentle stirred to get homogeneous hMSC suspensions. The bioinks were then stored at different temperatures (4 °C, 25 °C, or 37 °C) for further studies. Identical process of formulation, without hMSC, was performed to obtain a blank AL/HA-ink to be used as a control. Finally, the hMSC/AL/HA-bioink was placed in the bioprinter (REG4LIFE by Regemat; Regemat 3D, S.L., Granada, Spain) and extruded at room temperature (RT) (Flow velocity: 4.5 mm/s; nozzle diameter: 0.4 mm). After bioprinting, the crosslinking process was completed by bath in 180 mM CaCl_2_ for 30 min at RT. Then, the constructs were placed in culture medium (DMEM containing, 10% FBS and 1% streptomycin/penicillin) and stored for 7 days in an incubator with 5% CO_2_ at 37 °C.

### Sterility treatment and testing

The sterility assay was performed by direct inoculation of 1 mL AL/HA-bioink in two different microbiology media according to the European Pharmacopoeia 9th Edition, by using Thioglycollate Penase Broth (TPB; VWR Int., Radnor, PA, USA) to detect anaerobic and aerobic microorganisms, and Tryptic Soy Penase Broth (TSPB, VWR Int., Radnor, PA, USA) to detect fungi and aerobic microorganisms. Each hydrogel in inoculated media was incubated for 14 days at 22 °C and 35 °C for TSPB and TPB, respectively. All samples were daily inspected, by visually checking for any signs of turbility. If after 14 days microbial growth had taken place, the medium would show turbidity. Aseptic conditions were implemented for this assay, inside a safety cabinet in a clean room. For each media (TSPB and TPB), sterility test and growth promotion test of aerobes, anaerobes and fungi, were previously performed. The formulation and bioprinting processes were carried out under aseptic conditions to maintain the sterility of the final bioink and construct.

### Physical and macroscopic appearance

The gelation of AL/HA-hydrogel was assayed by tube inversion test, at three different crosslinking states, namely, non-crosslinked hydrogel, semi-crosslinked hydrogel (adding 100 mM CaCl_2_), and fully crosslinked hydrogel (with 180 mM CaCl_2_). The test tube inversion method allows determining the sol or gel state of a hydrogel depending on the flow of the sample. Briefly, the hydrogel was deposited on small-sized glass jars and immersed in a water bath at 37 ± 0.5 °C. To determine the sol-to-gel transition of the hydrogel, the jars were then inverted and the time to stop flowing freely was then recorded. The physical and macroscopic appearance of hMSC/AL/HA-bioink and AL/HA-ink were checked.

### Osmolality

Osmolality was determined in a micro-osmometer model 3320 (Advanced Instruments, Inc., Norwood, MA, USA) at 25 ± 0.5 °C. The freezing point depression (FPD), which refers to the direct proportional concentration of osmotically active compounds in aqueous solution, was determined. All measurements were performed in triplicate, and results are given as the mean ± standard deviation (SD).

### pH values

The pH values of bioinks were determined using a calibrated pH meter LAQUA pH1100-S (HORIBA Advanced Techno Co., Lille, France) at 4 ± 0.5 °C, 25 ± 0.5 °C and 37 ± 0.5 °C. Each measurement was conducted by direct immersion of the electrode in the samples contained in a glass. All measurements were run in triplicate, and results are given as the mean ± standard deviation (SD).

### Degradation test

The degradation test was undertaken by immersion procedure. Accurate weights of hMSC/AL/HA-bioink and AL/HA-ink (0.1 ± 0.02 g) were immersed in an Eppendorf tube with 1 mL PBS (pH 7.4). The test was performed at three distint temperatures, i.e., 4 ± 0.5 °C, 25 ± 0.5 °C and 37 ± 0.5 °C and at two different relative humidity (RH) values, 35% and 70% in a climatic chamber during 300 min. Eppendorf tubes tube were left close during the incubation. After incubation, samples were centrifuged at 5000 rpm for 1 min. The PBS excess was removed and the samples were weighed. Upon each analysis, 1 mL of PBS was added to keep original conditions constant. The degradation percentage was determined in triplicate as a measure of weight loss (WL) using the following equation [45]:

$$WL\;\left(\%\right)=\frac{W_0-W_t}{W_0}\;\times\;100$$

where W_0_ is the initial weight of sample and W_t_ the mass of degraded sample measured at different times.

### Swelling test

The water uptake capacity of hMSC/AL/HA-bioink and AL/HA-ink was determined by swelling of freeze dried samples in an Eppendorf tube with 500 µL PBS (pH = 7.4). The test was carried out at 4 ± 0.5 °C, 25 ± 0.5 °C and 37 ± 0.5 °C, and two different RH values, 35% and 70%, in a climatic chamber during 180 min. Eppendorf tubes tube were left close during the incubation. At selected time intervals the swollen samples were centrifuged at 5000 rpm for 1 min, and after eliminating the excess of PBS, they were weighed [45]. Then, samples were replenished with fresh PBS. The swelling (Sw) percentage was calculated in triplicate applying the following equation:

$$Swelling\;ratio\;\left(\%\right)=\frac{W_s-W_d}{W_s}\;\times\;100$$

where W_s_ is the weight of the samples at the swelling state and W_d_ is the initial mass of the freeze dried samples.

### Porosity

The porosity of hMSC/AL/HA-bioink and AL/HA-ink was calculated according to the Archimedes’ principle [46]. Analysis of percentage of porosity was performed by immersing the freeze-dried bioinks (0.06 ± 0.01 g) in 200 mL PBS pH 7.4 at 25 ± 0.5 °C. The submerged mass of samples was recorded, and then samples were removed, and the wet mass was recorded. Samples were analysed in triplicate. Porosity of bioinks was calculated applying the following equation:

$$Porosity\;\left(\%\right)=\frac{M_w-M_D}{M_w-M_{SUB}}$$

where M_w_ is the water saturated wet mass of samples, M_D_ is the freeze dried mass of the samples, and M_SUB_ is the submerged mass of samples.

In addition, AL/HA-ink and hMSC/AL/HA-bioink images obtained by environmental scanning electron microscopy (SEM; ESEM Quanta 200 FEI) were analyzed. The images were processed using ImageJ software following the procedure described by Monticeli et al. [47]. Briefly, the measurement scale was adjusted taking into account the values provided by the microscope. Then, the images were transformed to an 8-bit format and the threshold adjustment was carried out in the range 0-255 to be able to measure gaps in the sample and to set the appropriate threshold. In this case, the optimal range used was 0-115.

### Zeta potential and electrical conductivity

The zeta potential (ζ) of hMSC/AL/HA-bioink and AL/HA-ink was determined at 25 ± 0.5 °C from the electrophoretic mobility using a Zetasizer Nano ZS (Malvern Instruments Ltd., Malvern, UK). The electrical properties of samples were tested as a function of pH variation (from 4 to 8, at constant concentration 10^−3^ M KNO_3_), and as a function of ionic strength variation (from 10^−1^ to 10^−5^ M KNO_3_, at pH = 6). Before measuring, the samples were dispersed in double distilled water to form a 0.1% (w/v) solution, kept under mechanical stirring (50 rpm) for 24 h [48]. Zeta potential values were recorded as the mean ± SD of three replicates. Electrical conductivity of the samples was recorded in a Crison EC-Meter BASIC 30+ (Crison Instruments, Alella, Spain) conductivity meter. The samples were diluted in water at room temperature (1:20). The device electrode was dipped into a glass vial containing samples. Data are expressed as the mean ± SD of three replicates.

### Rheological studies

Samples of hMSC/AL/HA-bioink and AL/HA-ink were submitted to dynamic (oscillatory) test, as well as rotational test by using Haake RheoStress 1 rheometer (Thermo Fischer Scientific, Kalsruhe, Germany) connected to a temperature controller (Thermo Haake Phoenix II + Haake C25P). The initial rotational testing consisted of a stress sweep test followed by a frequency sweep test were carried out for the hMSC/AL/HA sample to determine the appropriate values of shear stress and frequency for the subsequent temperature sweep test. Each sample was set to equilibrate by placing it between the plate-plate sensor systems with a Haake PP60Ti mobile plate (1 mm gap, 60 mm diameter) for 5 minutes to reach the starting temperature. The oscillatory stress sweep tests were conducted at a constant frequency of 1 Hz and a temperature of 25 ± 0.5 °C, in a stress range of 0.04 to 200 Pa. A value of 0.5 Pa, located within the linear viscoelastic region, was selected for the subsequent frequency sweep study ranging from 0.1 to 10 Hz. Consequently, the effect of temperature on the rheology of the bioinks was investigated by performing a temperature sweep test from 10 ± 0.5 °C to 40 ± 0.5 °C at 1 Hz and 0.5 Pa. The temperature increment was controlled at a ramp speed over 2000 seconds, during which the storage modulus (G’), loss modulus (G’’) and the complex viscosity (η*) were measured.

In the second case, rotational tests were run in a Haake C60/2Ti mobile cone (60 mm diameter, 0.102 mm gap and 2 ° angle). Each sample was set to equilibrate by placing it between the cone-plate sensor systems (0.102 mm gap) for 5 min to attain the running temperature. The shear rate was increased from 1 to 100 s^-1^, then maintained for 1 min at 100 s^-1^, and finally decreased from 100 to 1 s^-1^. The shear stress was measured at 25 ± 0.5 °C and 37 ± 0.5 °C.

Data from the flow curve (τ = f (γ̇)) were fitted to selected mathematical models, namely, Bingham, Casson, Cross, Herschel-Bulkley, Newton and Ostwald-De-Waele, to determine the flow type. Viscosity mean values (Pa·s) were determined from the constant share range at 100 s^-1^ of the viscosity curve (η = f (γ) in response to the temperature (ºC). During the test, the microstructure disturbance or apparent thixotropy (Pa/s) was evaluated by calculating the area of hysteresis loop formed when the shear program is run, i.e., from 1 to 100 s^-1^ (ramp-up), constant shear at 100 s^-1^, and from 100 to 1 s^-1^ (ramp-down).

### Stability tests

Freshly prepared samples (*n* = 3) were submitted to heating-cooling cycles over a temperature range between 4 ± 0.5 °C and 45 ± 0.5 °C by storing them 24 h under each temperature for 6 days, and then checked for signs of physical instability (gel collapse, homogeneity, etc.). Freshly prepared samples (*n* = 3) were centrifuged (Centrifuge 5804 Eppendorf, Hamburg, Germany) at 3500 rpm for 30 min at room temperature (*n* = 3), and then examined for signs of physical instability. Freshly prepared samples went through freeze-thaw cycles, by keeping them between − 21 ± 0.5 °C and + 25 ± 0.5 °C, for three cycles of 24 h for 6 days, and then examined for sings of physical instability. Finally, the influence of the temperature on hMSC/AL/HA-ink was studied to define its appropriate temperature of storage and its shelf-life. Bioinks loaded with hMSC were packed in 3 cc Luer-Lock syringes at a concentration of 1 × 10^6^ cells/mL with 0.5 mL of AL/HA-bioink. Different temperatures were assayed 4 °C, 25 °C and 37 °C (three syringes each temperature). Cell viability was tested every 24 h for 3 days using the alamarBlue^®^ (aB) assay following the manufacturer’s protocol (Thermo Fisher Scientific Inc., Carlsbad, CA, USA).

### Scanning electron microscopy (SEM)

Cell-free and hMSC/AL/HA-bioinks were maintained under standard cell culture conditions at 0, 1, 2 and 3 days. Bioinks were fixed in a bath of 2.5% glutaraldehyde buffer in cacodylate for 12 h followed by a dehydration process through a graded series of ethanol (30–100%). Then, bioinks were critical-point dried in an Emscope CPD 750 critical point dryer (Emscope Lab., Ashford, UK). Bioinks were placed onto aluminum SEM specimen mounting stubs (Electron Microscopy Sciences, Hatfield, PA, USA) on a carbon tab (Agar Scientific Ltd., Essex, UK) and coated with carbon to improve their electrical conductivity using an Emitech^®^ K950X Carbon Evaporator (QuorumTechnologies Ltd, East Grinstead, UK). Finally, samples were examined using a field emission scanning electron microscope JSM-7001 F (JEOL Ltd., Tokyo, Japan). Morphology and porosity of critical-point dried hMSC/AL/HA-ink were observed and measured respectively by SEM.

### Optical microscopy

To determine whether the hMSC were properly embedded and distributed along the bioinks, cells were first examined by optical microscopy at 0 and 7 days. Images were taken using a microscope (Olympus IX71, Tokyo, Japan) at 100× magnification.

### Cell viability

The LIVE/DEAD^®^ Viability/Cytotoxicity kit (Thermo Fisher Scientific Inc., Carlsbad, CA, USA) was used following the manufacturer’s instructions to assess cell viability in hMSC/AL/HA-bioinks. Briefly, samples were firstly washed with 1X PBS wash buffer. Subsequently, they were incubated in the dark for 30 min with 8 µL of 4 µM EthD-I (red staining) and 4 µL of 2 µM calcein AM (green staining), both diluted in 4 mL sterile PBS. Following another wash with 1X PBS, samples were observed using a confocal microscope Nikon Eclipse Ti-E A1 (Nikon Instruments Europe B.V., Amsterdam Netherlands) and imaged on days 1, 4 and 7. Green fluorescence was indicative of live cells while red fluorescence indicated dead cells using two distinct filters. The images were then analyzed using ImageJ software v. 1.52i (NIH, Bethesda, MD, USA). Six regions were assessed for each cell type (live or dead) to derive an average value of the percentage of viable cells (*n* = 3).

### Cell proliferation assay

The proliferation rate of cells embedded in hMSC/AL/HA-ink was determined by colorimetric aB assay (Thermo Fisher Scientific Inc., Carlsbad, CA, USA) on day 1, 4 and 7. Appropriate control without cells was used for data normalization. Briefly, samples were incubated with 10 µL of aB solution per each 100 µL of medium at each time point, and incubated for 3 h. The fluorescence intensity was analysed using a plate reader Synergy HT (Bio-Tek Instruments, Inc., Winooski, VT, USA) with excitation and emission wavelengths of 600 nm and 570 nm. Experiments were performed in triplicate (*n* = 3), and the absorbance data represent as fold increase to day 1.

### 3D bioprintability

An extrudability test was performed using hMSC/AL/HA-ink in three different crosslinking states (0 mM, 100 mM and 180 mM CaCl_2_) to determine the appropriate degree of crosslinking for use in bioprinting. It was done by varying bioprinting properties such as flow rate or printing speed [5, 49–51].

The selected hMSC/AL/HA-ink was packed in a 3 cc syringe and loaded into the bioprinter. The bioprintability assessment of the bioink was performed using a REG4LIFE by Regemat bioprinter (Regemat 3D, S.L., Granada, Spain). The bioprinting process consisted of the deposition of the selected bioink in a layer-by-layer manner adding 180 mM CaCl_2_ to generate a linear 3D printed fully-crosslinked construct (porous rectangular-type structure; 10 mm high ×10 mm wide structure) by using the Regemat 3D designer software [52]. Filament after extrusion and nozzle diameter had a width of 0.4 mm. The adequate flow speed was established at 4.5 mm/s. Furthermore, to determine whether the bioprinting process of the cell-loaded bioink affected cell proliferation capacity, an aB assay was performed post-bioprinting at 0 h and 24 h.

### Statistical analysis

The obtained results were statistically validated by one-way analysis of variance (ANOVA) or Student’s *t*-test using GraphPad Prism^®^ v. 5.00 software (GraphPad Software Inc., San Diego, CA, USA). Differences between independent groups were determined by applying Tukey’s test, the significance level was set at 0.05, and adopting a 95% confidence level.

## Results

### Bioink formulation

The developed bioink was formulated from a non-crosslinked AL/HA-hydrogel. Since all biomaterials used for biomedical applications must be sterile, before to formulation of non-crosslinked hydrogels, AL and HA powder were exposed to ultraviolet (UV) radiation overnight prior to their use. UV radiation has been previously shown to be effective in the sterilization of materials, such as HA, against some types of bacteria without affecting their molecular structure [53, 54]. To evaluate whether the non-crosslinked AL/HA-hydrogels were sterile before to add hMSC, a sterility test was conducted, by incubating a sample of AL/HA-hydrogel in TSB and TSPB. After 14 days of incubation, the incubated culture media showed no turbidity, which was indicative of no microbial growth, concluding that developed non-crosslinked AL/HA-hydrogel was sterile. Then, this hydrogel was semi-crosslinked in presence of calcium thought physical crosslinking process. Afterwards, semi-crosslinked AL/HA-hydrogels were loaded with hMSC and packed, obtaining syringes loaded with the hMSC/AL/HA-bioink.

### Physicochemical characterization

The gelation process of AL/HA-hydrogel was studied at three different crosslinking states by tube inversion test. This test demonstrated that as the CaCl_2_ concentration increased, the hydrogel required shorter gelation time. Thus, the hydrogel without CaCl_2_ never jellified while the semi-crosslinked hydrogel needed approximately 56.67 ± 15.28 s to generate a gel. The fully crosslinked hydrogel needed only 33.33 ± 5.77 s (*n* = 3) resulting a non-flowable hydrogel. Therefore, the semi-crosslinked hydrogel was selected to formulate the bioink, increasing the handling time and avoiding gelation problems at the tip of the syringe. After the formulation and semi-crosslinking process, the hMSC/AL/HA-bioink and AL/HA-nk presented homogenous appearance and transparent color.

The results of pH values of bioinks are depicted in Table 1. The pH values of AL/HA-ink at 4 ± 0.5 °C and 25 ± 0.5 °C slightly increased from 0 h to 72 h, whereas the pH values of AL/HA-ink at 37 ± 0.5 °C slightly decreased over time. No statistically significant differences between bioinks at different temperature values were observed (*p* > 0.05).

**Table 1:** Results of pH values recorded for AL/HA-ink and hMSC/AL/HA-bioink at different temperature conditions (4 ± 0.5 ºC, 25 ± 0.5 ºC and 37 ± 0.5 ºC), and different time-points

**Time (hours)**	**4 °C**		**25 °C**		**37 °C**	
	**AL/HA-ink**	**hMSC/AL/HA-bioink**	**AL/HA-ink**	**hMSC/AL/HA-bioink**	**AL/HA-ink**	**hMSC/AL/HA-ink**
**0 h**	6.57 ± 0.31	6.54 ± 0.34	6.59 ± 0.46	6.61 ± 0.40	6.66 ± 0.33	6.69 ± 0.24
**24 h**	6.86 ± 0.22	6.57 ± 0.41	6.67 ± 0.34	6.61 ± 0.44	6.48 ± 0.47	6.52 ± 0.35
**48 h**	6.64 ± 0.35	6.59 ± 0.50	6.62 ± 0.29	6.62 ± 0.35	6.60 ± 0.39	6.60 ± 0.27
**72 h**	6.71 ± 0.21	6.72 ± 0.28	6.78 ± 0.18	6.62 ± 0.54	6.56 ± 0.28	6.62 ± 0.34

AL/HA-bioink showed low osmolality values. This result slightly increased after the addition of hMSC (Supplementary Table S1). Both values were hypo-osmotic regarding the physiological osmolality (i.e., 0.28 OsM/kg). Nevertheless, no negative effects on cell behavior were identified.

**Fig. 1:**
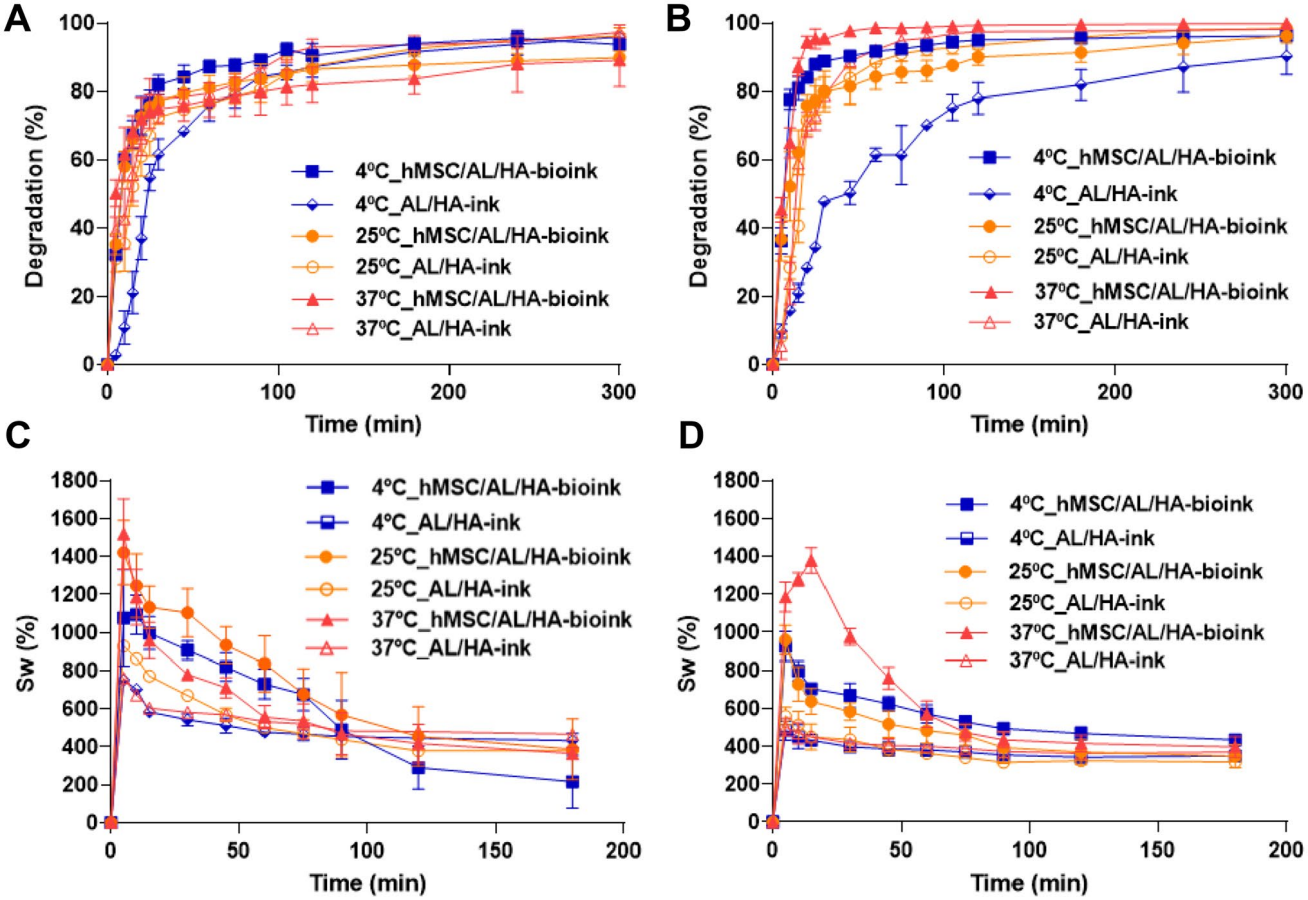
Degradation and swelling profiles of bionks. **A** Degradation percentage of bionks at pH 7.4 at 4 ± 0.5 °C, 25 ± 0.5 °C and 37 ± 0.5 °C and RH of 35% after 300 min; **B** Degradation percentage of bioinks at pH 7.4 at 4 ± 0.5 °C, 25 ± 0.5 °C and 37 ± 0.5 °C and HR of 70% after 300 min; **C** Swelling rate of bioinks at pH 7.4 at 4 ± 0.5 °C, 25 ± 0.5 °C and 37 ± 0.5 °C and RH of 35% after 180 min; **D** Swelling ratio of bioinks at pH 7.4, 4 ± 0.5 °C, 25 ± 0.5 °C and 37 ± 0.5 °C and RH of 70% after 180 min. Values represent mean ± SD (*n* = 3)

The degradation of AL/HA-ink and hMSC/AL/HA-bioink was evaluated as the percentage of weight loss (WL) at different temperatures and relative humidity (RH) conditions. As shown in Fig. 1A, after 300 min, at 35% RH the AL/HA-ink and hMSC/AL/HA-bioink exhibited degradation rates of 96.03 ± 1.19% and 93.73 ± 0.39%, respectively (4 ± 0.5 °C); 96.15 ± 2.52%, 89.88 ± 1.88%, respectively (25 ± 0.5 °C); and 97.32 ± 2.17%, 89.16 ± 7.76%, respectively (37 ± 0.5 °C). During the first 60 min, the degradation of AL/HA-ink was slower than of hMSC/AL/HA-bioink. However, AL/HA-ink showed a degradation value higher than hMSC/AL/HA-bioink after 300 min. In AL/HA-bioink, it was also observed a slight increase on degradation rate when temperature increased. On the other hand, for hMSC/AL/HA-bioink an increase on degradation rate was seen when temperature decreased. As shown in Fig. 1B, after 300 min and 70% RH, degradation rates of AL/HA-ink and hMSC/AL/HA-bioink were 90.39 ± 5.40% and 96.28 ± 0.27%, respectively (at 4 ± 0.5 °C); 98.41 ± 1.05%, 96.26 ± 2.10%, respectively (at 25 ± 0.5 °C); and 98.26 ± 1.60%, 99.89 ± 0.20%, respectively (at 37 ± 0.5 °C). These results also showed that hMSC/AL/HA-bioink exhibited a degradation value higher than AL/HA-ink after 300 min. During the first 60 min, the degradation of AL/HA-ink was slower than hMSC/AL/HA-bioink. These results highlight how degradation rate increased with temperature in AL/HA-ink. However, hMSC/AL/HA-bioink at 25 ± 0.5 °C presented the lowest degradation rate, but the fastest degradation rate was observed at 37 ± 0.5 °C. These results suggest that AL/HA-ink was degraded more quickly at 35% RH than at 70% RH, whereas hMSC/AL/HA-bioink was degraded more quickly at 70% than at 35% RH.

Results of the swelling test are depicted in Fig. 1C and D. The hMSC/AL/HA-bioink exhibited higher swelling ratios compared to AL/HA-ink at 35% RH and same temperature. The rate of maximum swelling of AL/HA-ink at 4 °C, 25 °C and 37 °C was reached after 5 min, 751.58 ± 26.67%, 929.77 ± 1.98% and 761.95 ± 31.36%, respectively. The hMSC/AL/HA-bioink reached the maximum swelling after 10 min at 4 ± 0.5 °C (1094.74 ± 104.52%), after 5 min at 25 ± 0.5 °C (1094.74 ± 104.52%) and 37 ± 0.5 °C, (1519.92 ± 185.63%). It can also be seen how the bioinks started to lose weight and seem to stabilize over time. The lowest swelling rate was obtained at 4 °C for both bioinks.

Figure 1D shows swelling behavior of bioinks at 70% RH. The rate of maximum swelling of AL/HA-ink at 4 ± 0.5 °C (483.56 ± 50.63%), 25 ± 0.5 °C (560.42 ± 46.08%) and 37 ± 0.5 °C (502.74 ± 45.54%) was reached after 5 min. The maximum percentage of swelling of hMSC/AL/HA-bioink was reached after 5 min at 4 ± 0.5 °C (926.73 ± 78.63%) and 25 ± 0.5 °C (959.64 ± 78.22%) and after 10 min at 37 ± 0.5 °C (1278.20 ± 36.17%). When comparing both at the same temperature, hMSC/AL/HA-bioink exhibited higher swelling capacity than AL/HA-bioink.

The percentage of porosity was determined by immersing the AL/HA-ink and hMSC/AL/HA-bioink in PBS pH 7.4 at room temperature. The porosity was found to be 48.89 ± 7.38% for AL/HA-ink and 52.30 ± 4.26% for hMSC/AL/HA-bioink. The difference in porosity between AL/AH-ink and hMSC/AL/AH-bioink is not statistically significant. To validate these results, SEM images of hMSC/AL/HA-bioink and AL/HA-ink were also analyzed to check the percentage porosity of the inks using the image thresholding method of ImageJ software (Supplementary Fig. S1). The pixels highlighted in red are classified as either empty or part of the porosity. Subsequently, we conducted a quantitative analysis of the image to determine the percentage of colored area. This analysis indicated a porosity value of 44.89 ± 14.06% in hMSC/AL/HA-bioink and 44.97 ± 17.69% in AL/HA-ink.

**Fig. 2:**
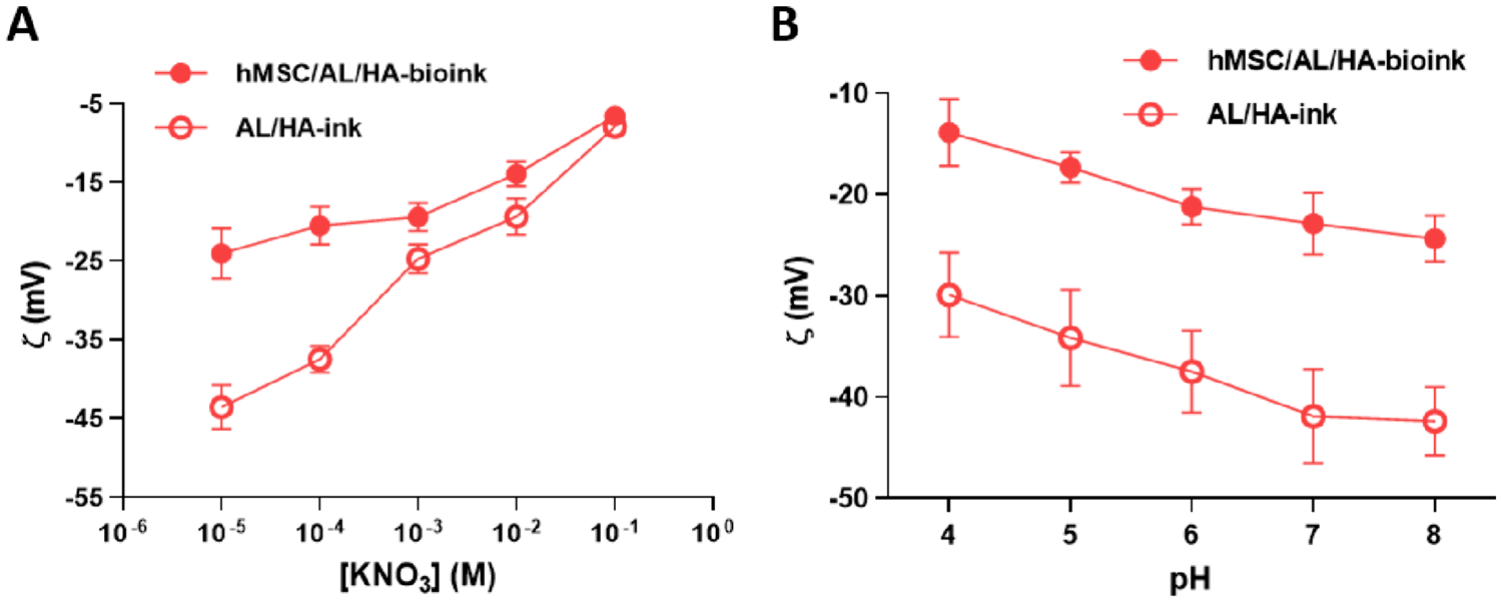
Zeta potential (ζ, mV) of bioinks. **A** AL/HA-ink and hMSC/AL/HA-bioink as a function of ionic strength at pH = 6 at 25 ± 0.5 °C; **B** as a function of pH in the presence of 10^−3^ M KNO_3_ concentration at 25 ± 0.5 °C. Values represent the mean ± SD (*n* = 9)

Measurements of zeta potential are shown in Fig. 2. A negative zeta potential value was noticed in the assayed ionic strength range for both samples. Furthermore, the zeta potential showed a dependence on the ionic strength (Fig. 2A). Absolute value of zeta potential decreased when the KNO3 concentration increased. The obtained results of zeta potential ranged from -7.95 ± 1.19 mV to -43.56 ± 2.81 mV, and from -6.57 ± 0.80 mV to -24.02 ± 3.17 mV for AL/HA-ink and hMSC/AL/HA- bioink, respectively.

A negative zeta potential was also noticed for the assayed pH range. Figure 2B shows the zeta potential values as a function of the pH in the presence of 10-3 M KNO3 at 25 °C. The zeta potential showed a dependence on pH. The zeta potential (in absolute value) followed an increase as pH increased, from -29.88 ± 4.18 mV to -42.41 ± 3.38for AL/HA-ink and from -13.85 ± 3.28 mV to -24.34 ± 2.29 mV for hMSC/AL/HA-bioink, respectively. Our results showed a negative zeta potential in the assayed ionic strength and pH range owing to the presence of carboxyl and hydroxyl groups on the surface of AL and HA. The zeta potential gives a broad idea about the surface chemistry of a material, with significant differences between the developed bioinks. The obtained values suggest that some cells are distributed near the surface of bioink and, for this reason, the zeta potential decrease (in absolute value). Conductivity values for AL/HA-ink and hMSC/AL/HA-bioink were 1790.01 ± 2.29 µS/cm and 1321.02 ± 3.82 µS/cm, respectively.

**Fig. 3:**
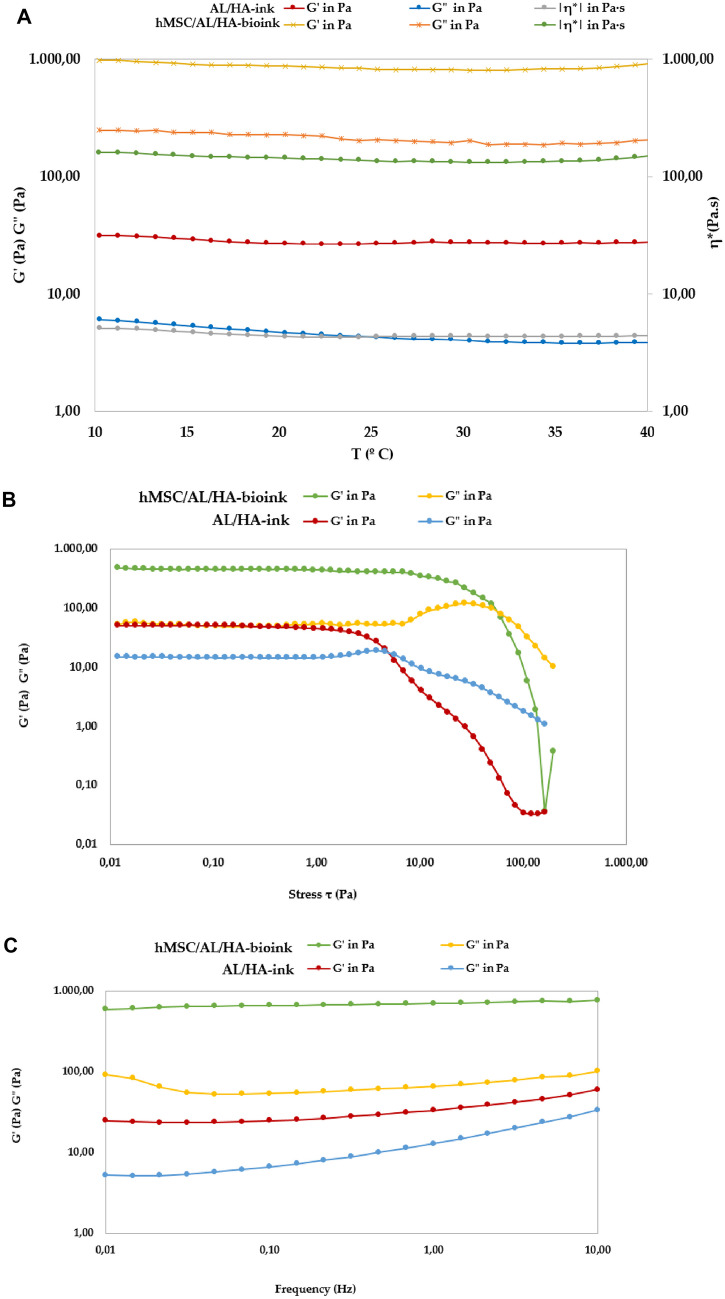
Rheological studies of bioinks. **A** Storage modulus (G’), loss modulus (G’’) and complex viscosity (η*) versus temperature, of AL/HA-ink and hMSC/AL/HA-bioink. **B** Results of amplitude oscillatory sweep and **C** results of frequency oscillatory sweep for AL/HA-ink and hMSC/AL/HA-bioink

The AL/HA-ink and hMSC/AL/HA-bioink presented the same aspect of a thick hydrogel at both low and high temperatures and, thus, both were characterized as predominantly elastic (G’ > G’’) (Fig. 3). The oscillatory stress sweep with samples of hMSC/AL/HA bioink were performed at a constant frequency of 1 Hz and 25 ± 0.5 °C in a stress range from 0.01 to 200 Pa. In these testing conditions, the critical stress appeared near 1 Pa (Fig. 3B). The subsequent frequency sweep study between 0.1 and 10 Hz at 0.5 Pa (Fig. 3C) did not show significant changes in the G’ and G’’ moduli for hMSC/AL/HA-bioink. Therefore, the temperature sweep test was conducted at 1 Hz and 0.05 Pa. The results confirm that both bioinks were not thermosensitive and, when the temperature increased from 10 °C to 40 °C, the values of both moduli (G’ and G’’) remained reasonably constant (Fig. 3A). However, both bioinks exhibited differences in their viscoelastic properties as the values of G’, G’’ and 𝜂* were clearly smaller in the case of AL/HA-ink.

**Fig. 4:**
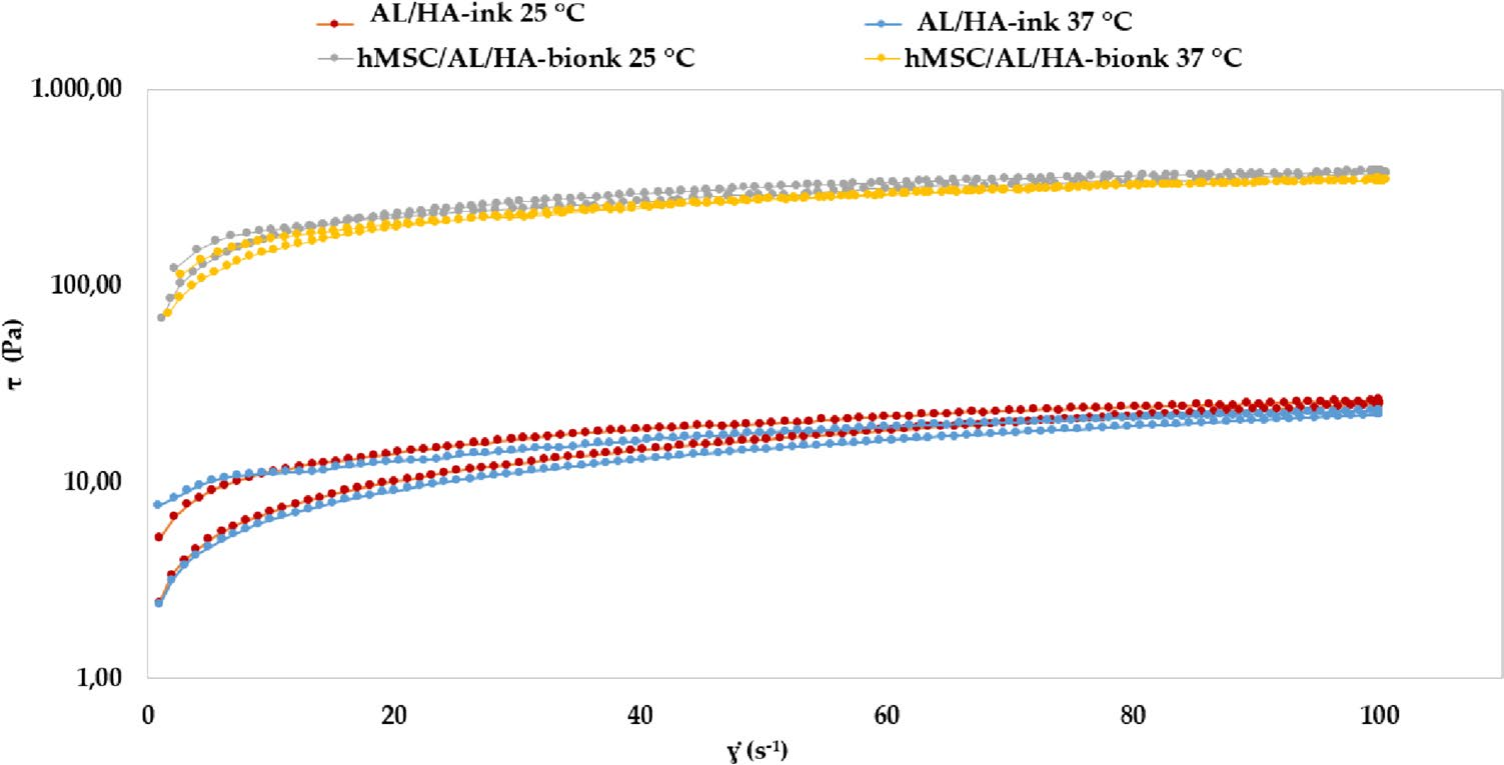
Shear stress (τ) versus shear rate (ɣ̇) bioinks at 25 ± 0.5 °C and 37 ± 0.5 °C

Figure 4 displays de flow curves of bioinks, which were obtained from the rotational rheology studies. AL/HA-ink and hMSC/AL/HA-bioink revealed a non-Newtonian profile adjusting to shear thinning flow at 25 ± 0.5 °C and 37 ± 0.5 °C.

The adjusted mathematical model for the flow curves, as well as the viscosity values and aparent thixotropy of bioinks, are provided in the Supplementary Table S2. Viscosity results (at 100 s^-1^) showed a higher value for hMSC/AL/HA-bioink. Additionally, the profiles of the viscosity curves at 25 ± 0.5 °C and 37 ± 0.5 °C were quite similar (Supplementary Fig. S2). The remarkable increase in the area of hysteresis loop, and of the viscosity of the hMSC/AL/HA-bioink compared to AL/HA-ink, confirms that the addition of cells provides a higher degree of structuration. Other authors reported that bioinks with viscosity range of 0.30–30 Pa·s were suitable for extrusion bioprinting [55]. Moreover, this study suggests that a material with a viscosity below 0.3 Pa.s is more suitable for smearing rather than printing and if the viscosity of the material is higher than 300 Pa.s; high pressure is required to extrude the hydrogel from the nozzle, making the extrusion process unstable [55]. According to this range of viscosity, our hMSC/AL/HA-bioink could be appropriate for extrusion-based bioprinting.

After subjecting to healing-cooling cycle, centrifugation cycle and freeze-thaw cycle, AL/HA-ink did not exhibit any sign of instability (Supplementary Fig. S4). All these results confirmed that developed bioinks can provide optimal support for cells avoiding potential structural disruption.

**Fig. 5:**
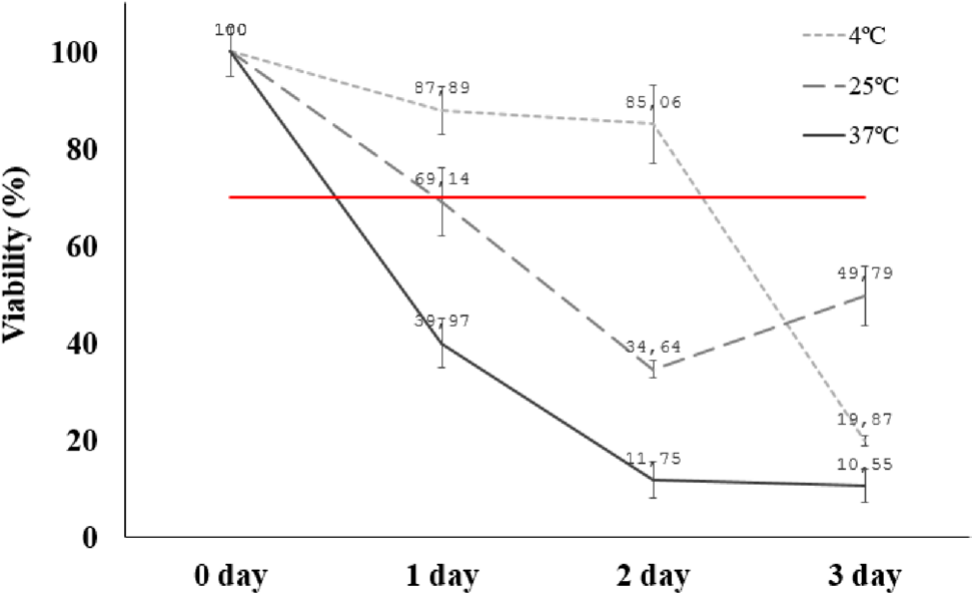
Viability of hMSC embedded in AL/HA-bioinks packed in 3 cc syringes and stored at 4 ± 0.5 °C, 25 ± 0.5 °C and 37 ± 0.5 °C for 3 days. Each point represents mean ± SD (*n* = 3). The red line represents the threshold line above which the percentage of viability can be considered adequate (70%)

A pre-bioprinting bioink preservation and storage study was conducted to determine the shelf-life of the bioink once loaded into the 3 cc syringes without basal cell culture medium that supports cell growth. The goal of this stability assay was to determine the maximum time frame that our bioink can be preserved and what storage temperature would be most appropriate to maintain viability above the minimum acceptable 70%. The average viability of the hMSC cultured before being embedded in the bioink was 99.81 ± 1.10%. Then, every 24 h the cell viability of each sample was assessed. The results showed that after one day, the viability of hMSC decreased considerably at 37 ± 0.5 °C (39.70 ± 10.03%) compared to samples stored at 4 ± 0.5 °C and 25 ± 0.5 °C (87.90 ± 9.80%, and 70.0 ± 13.83%, respectively), in which cell viability remained higher than 70%. The decrease in cell viability can be attributed to a combination of different factors, such as oxygen exchange to the embedded cells, lack of nutrients since the ink does not have cell culture medium with factors as glucose in its composition [56], and the storage temperature affecting the metabolic activity of the cells [57]. On the second day, only the hMSC/AL/HA-bioink stored at 4 ± 0.5 °C kept an optimal cell viability (85.10 ± 15.9%). Thus, this temperature was the most suitable for maintaining cell viability for longer time, since samples stored at 25 ± 0.5 °C and 37 ± 0.5 °C showed values lower than 70% (34.64 ± 3.53% and 11.75 ± 12.30%, respectively). Finally, on the third day, the percentage of cell viability decreased for all tested temperatures, namely, 19.87 ± 2.13% (4 ± 0.5 °C), 49.49 ± 12.31% (25 ± 0.5 °C) and 10.55 ± 6.91% (37 ± 0.5 °C) (Fig. 5).

### Scanning electron microscopy and optical microscopy

**Fig. 6:**
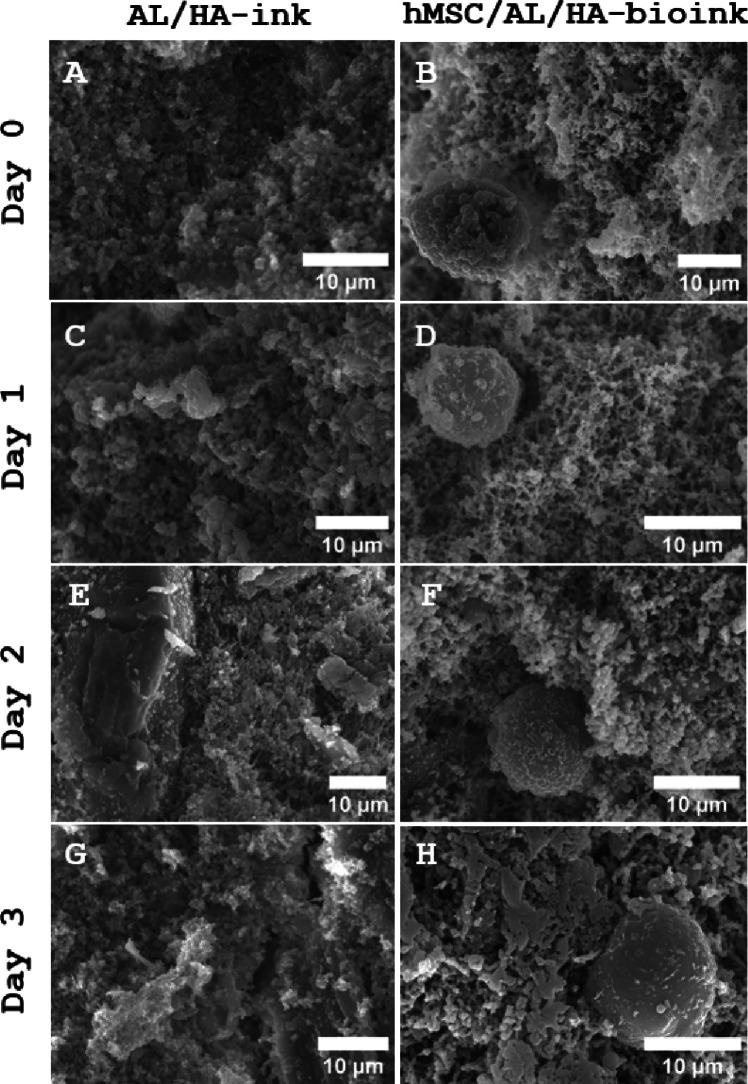
Scanning electron microscopy (SEM) images of critical point dried cell free AL/HA-ink and hMSC/AL/HA-bioink after 0, 1, 2 and 3 days. Magnification: 2,000× **E**, 2,200× **B**, 2,500× **C**, **G**, 3,000× **A**, **F**, 3,300× **H**, 3,500× **D**

As seen in Fig. 6, hMSC/AL/HA-bioink and AL/HA-ink showed porous structures of variable size and homogeneity. The morphology of the inks are microstructurally similar apart from the embedded cells that can be observed in hMSC/AL/HA bioink.

Optical morphology was also studied in the hMSC/AL/HA-bioink and AL/HA-ink (Supplementary Fig. S4) to verify if samples were homogeneous, and to check any cell aggregation in hMSC/AL/HA-bioink. Figure S3 shows the homogeneous morphology of AL/HA-bioink and hMSC/AL/HA-bioink. Images were taken on day 0 and day 7 of standard cell culture conditions with the optical microscope No cell aggregation was observed in hMSC/AL/HA-bioink, instead they were able to proliferate after 7 days of culture.

### Cell viability and proliferation

**Fig. 7:**
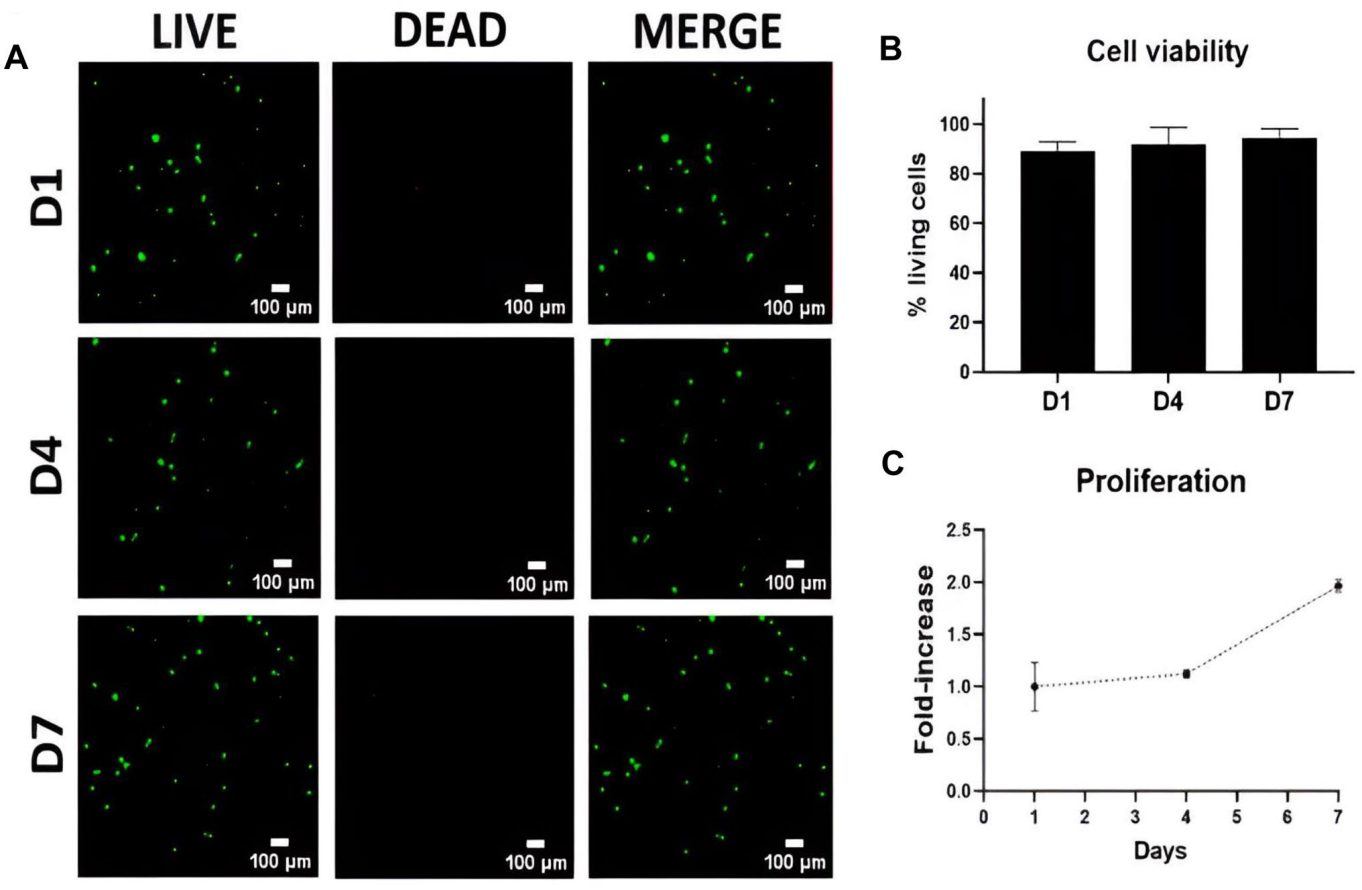
Cell viability of hMSC in hMSC/AL/HA-bioink. **A** hMSC in the AL/HA-bioink after one week culture, showing living cells (green fluorescence) and dead cells (red fluorescence); **B** percentage of hMSC viability in the AL/HA-bioink regarding culture time; **C** hMSC proliferation inside the AL/HA-bioink. Each point represents mean ± SD (*n* = 3)

Figure 7 shows confocal images from stained hMSC/AL/HA-bioink hydrogels over time, showing a uniform cell distribution, mostly exhibiting green fluorescence, which is indicative of cell viability (Fig. 7A). After the first day, there was 90% of living cells embedded in hMSC/AL/HA-bioink. The cell viability reached values of 92.8% after 4 days, reaching 94.5% after 7 days, although the differences were not statistically significant in comparison to the first day (Fig. 7B).

The cell proliferation of the hMSC/AL/HA-bioink was analyzed by aB assay. Figure 7C shows that hMSC embedded in the bioink exhibited an increase in cell population during the period of culture. After 4 days, the number of cells was 1.2 times higher when compared to the first day, and 2 times higher when compared to 7th day. The cell proliferation in hMSC/AL/HA-bioink was significant by the end of culture timeframe (7th day).

### 3D Bioprintability assessment

**Fig. 8:**
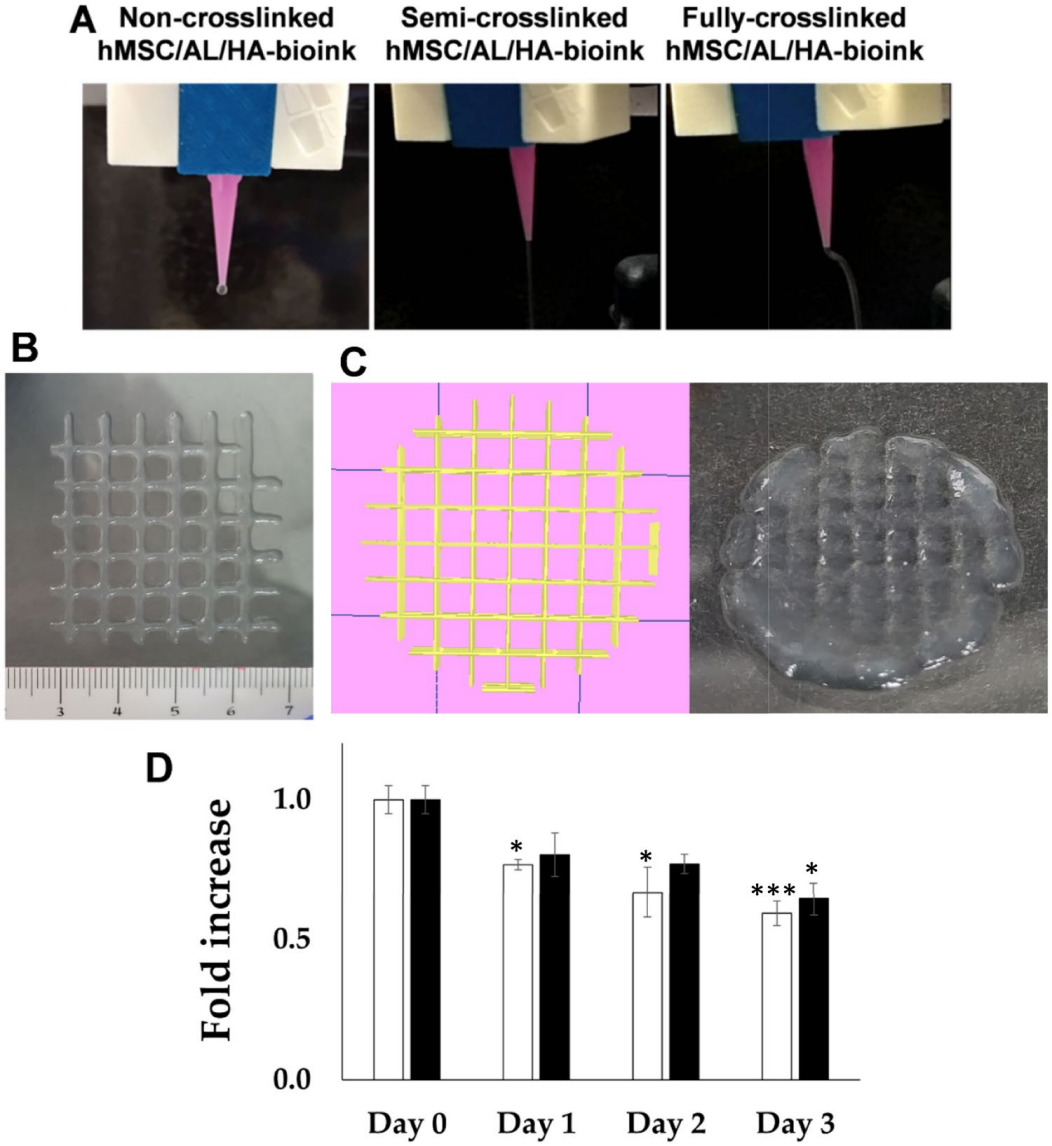
3D bioprintability of the hMSC/AL/HA-bioink. **A** Filament drop test at different stages. **B** Printability evaluation of an hMSC/AL/HA-bioink 2-layer construct, size 30 × 30 mm.; **C** On the left of the image, the computer-aided design of the constructs, size 10 × 10 mm. On the right side of the image the final semi-crosslinked construct. Filament width of 0.4 mm.; **D** Cell proliferation of cells embedded in hMSC/AL/HA-bioink, pre-bioprinting (white bars), and in the construct bioprinted by extrusion using the REG4LIFE by Regemat bioprinter, post-bioprinting (black bars). Data represent the mean ± standard deviation of the statistically significant difference between groups with respect to samples bioprinted on day 0 (*n* = 3). (*) *p* < 0.05; (**) *p* < 0.01; (***) *p* < 0.005

As seen in Fig. 8A, the non-crosslinked hMSC/AL/HA-bioink did not form a uniform filament. This sample was not able to maintain its shape after bioprinting process. The semi-crosslinked hMSC/AL/HA-bioink was able to form uniform filaments that maintain their shape. On the other hand, the fully crosslinked hMSC/AL/HA-bioink was not able to be properly bioprinted since it was too gelled. In this case, the extruded filament was non-uniform, which caused failure in the extrusion process and blockage of the nozzle.

**Fig. 9:**
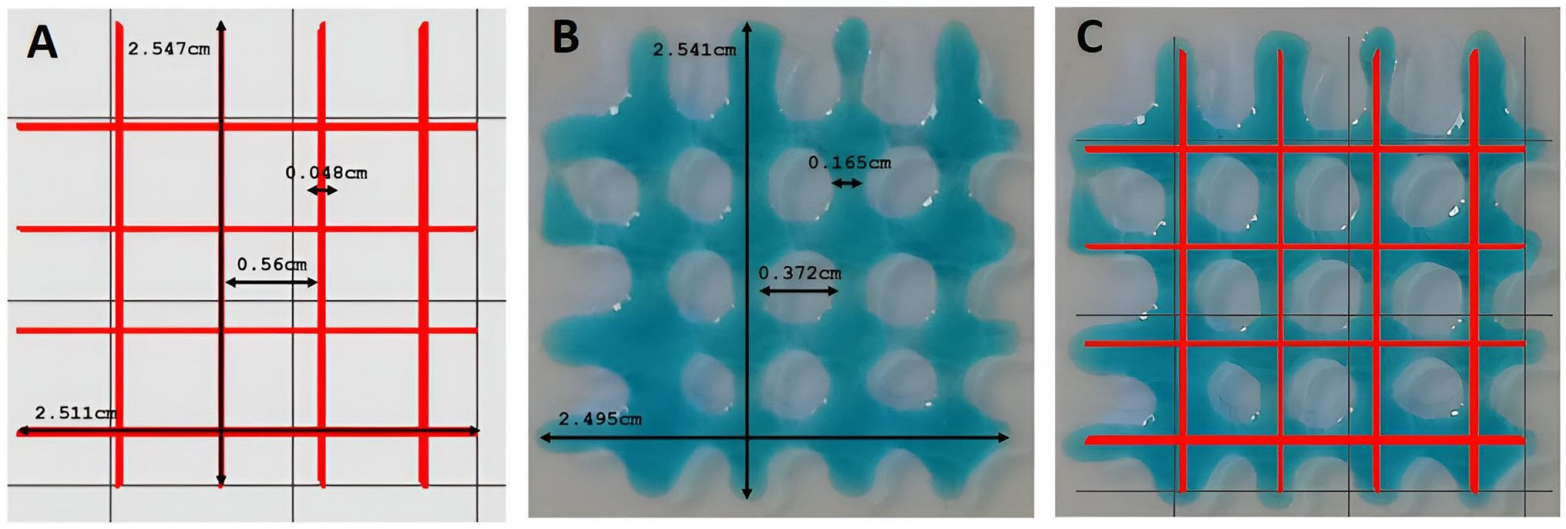
Quantitative analysis of shape fidelity. **A** Computer-aided design of the construct, size 25 mm x 25 mm. **B** Optical image of final bioprinted hydrogel, size 25 mm x 25 mm. **C** Image of the construct overlapped with its 3D computer-aided design model. In the preparation process of the hydrogel, AL/HA-ink was augmented with blue food coloring to facilitate measurements on the model

After selecting the semi-crosslinked bioink as the best condition for extrudability, the filament shape was checked (Fig. 8B). The hMSC/AL/HA-bioink was linearly deposited, generating a layer-by-layer printed model (porous structure of the rectangular type; 30 × 30 mm) previously designed with the Regemat 3D design software. The layer height of the filament was 0.2 mm, and nozzle diameter had a width of 0.4 mm. The adequate flow speed was established at 4.5 mm/s and the infill speed was set at 10 mm/s. The accuracy of the bioprinting process was obtained by comparing the area of the bioprinted constructs with the initial computer-designed surface (30 mm^2^). Quantitative analysis of shape fidelity was carried out [58] (Fig. 9). Slight variations in the shape of the bioink once extruded were observed.

Figure 8C shows the computer-aided design of the final hydrogels and the result obtained after the bioprinting process. In this case, cylindrical hydrogels of 10 × 10 mm and pore size 1 × 1 mm were bioprinted. The remaining bioprinting parameters were maintained. As the semi-crosslinked bioink exhibited the best extrudability properties, the final crosslinking was done post-bioprinting using a second drip-operated bioprinter head by which a layer-by-layer CaCl_2_ bath at 180 mM was made [59]. To determine whether the bioprinting process affected cell proliferation capacity of the cells, a post-bioprinting colorimetric aB assay was performed in the construct at 0 h, 24 h, 48 h and 72 h. The results showed that hMSC were not affected by bioprinting process although they were affected over time (Fig. 8D).

## Discussion

Bioinks are bioprintable formulations which mainly combine biomaterials with living cells to be used in 3D bioprinting processes [60]. Bioinks must mimic the ECM environment to obtain an optimal structural support, favoring cell adhesion, proliferation and differentiation. Therefore, physicochemical, mechanical, and biological requirements must be considered to biofabricate an artificial construct with similar characteristics to native tissues or organs. The presence of living cells could alter the bioink characteristics, thus it is necessary to evaluate the bioink properties before and after living cell addition [4, 9]. In this study, the bioink was formulated from a hydrogel. The gel was prepared by dissolution of HA and AL in PBS combined with 1% of CaCl_2_ (semi-crosslinked AL/HA-hydrogel), and subsequently, the cell pellet was added to obtain the hMSC/AL/HA-bioink. HA-based hydrogels are often subject to rapid degradation and have poor mechanical properties. Crosslinked HA hydrogels have a dense network structure. The HA macromolecules are highly aggregated and locally folded. This makes them less susceptible to degradation and prolongs their half-life. The crosslinking concentration or density determines the physical properties of the hydrogel and the degree of crosslinking affects the degradation rate of the hydrogel. Physical crosslinking interactions offer several benefits for use as bioinks. However, they often exhibit inadequate mechanical strength due to the low potency of physical crosslinking interactions, and controlling their degradation rate proves challenging. Chemical crosslinking offers more options for preparing crosslinked networks than physical crosslinking. However, may cause cytotoxicity and immune responses in the host [61].

The molecular weight and distribution of biomaterials in the bioink may affect its properties of flow and of bioprintability [62]. Our bioink was made of high molecular weight HA, which has been widely reported to possess an excellent biocompatibility and cytocompatibility for a range of applications [63]. Furthermore, the use of HA of high molecular weight and increased concentration in polymer solutions leads to the reinforcement of the 3D network. High molecular weight HA solutions also show enhanced viscosity and progressively increased viscoelasticity. These conditions are essential for the bioprinting process [64]. The gelation process of the HA/AL-hydrogels was due to the variation in the degree of crosslinking by reaction of Ca^2+^ ions and sodium AL. Previous studies reported hydrogel gelation times up to 2 min [65]. Our results showed gelation times of the semi-crosslinked hydrogel below 1 min, which is an important advantage that allows an optimal extrudability. These results were confirmed through the shape fillament study and bioprintability of the hMSC/AL/HA-bioink. These results demonstrate an adequate density and consistency of bioink while maintaining the layer height and geometry of the bioink after bioprinting process. Finally, the calcium bath would be desirable to finish crosslinking the bioprinted construct to improve the mechanical properties.

From a physicochemical perspective, our bioinks had high porosity [66], swelling properties which allow the diffusion of nutrients and other molecules through the construct [67]. Similarly, the surface characteristics (zeta potential and conductivity) were suitable for cell-to-cell and cell-to-material interactions [46], besides the apropriate rheological properties for the futher bioprinting process [68]. Bioinks have a number of requirements they must fulfill: (i) they must be biocompatible and nontoxic to cells and not promote host immune responses if implanted and must provide bioactivity/cell attachment sites to allow for cell survival, attachment, and proliferation, (ii) they must have appropriate mechanical properties to withstand mechanical forces during handling and implantation, (iii) an appropriate swelling due to influences the final shape and the size of the printed 3D construct, (iv) an appropriate degradation characteristics, vi) relatively higher viscosity to maintain cell suspension homogeneously, vii) provide initial structural integrity and strong shear-thinning behavior to minimize shear stress-driven cell damage during the printing process, viii) rapid cross-linking process after printing [69]. Therefore, the physicochemical parameters of a hydrogel, including rheological behaviour, swelling properties, degradation properties, surface tension and gelation kinetics, are important factors to ensure successful extrusion-based bioprinting [9].

The osmolality is an essential condition for cell survival favoring the oxygen and nutrient diffusion [70]. The osmolality should be similar to the physiological value (around 300 mOsm/kg) because cells could die if they are re-suspended in non-physiological polymeric solutions. Our developed bioinks were hypoosmotic. Despite this, we obtained high cell viability into the bioink. These results are in agreement with previous works about bioinks for tissue engineering. Lafuente-Merchan et al. [42] developed hypoosmotic bioinks and demonstrated that low osmolality values did not affect the cell behavior.

The pH values of AL/HA-ink and hMSC/AL/HA-bioink remained unchanged during the 72 h of study. It has been established that a pH of 6.8-8 is acceptable for cell survival and viability [71]. Similar pH value was obtained by Lafuente-Merchan et al. [42]. These authors reported the development of an AL/HA-ink with pH value of 6.28 ± 0.03, and demonstrated that cells were alive after bioprinting process.

The degradation allows a progressive replacement of the construct with newly formed ECM of continuously growing cells under regeneration. The products of bioink should be fully degradable and should not induce inflammatory host response when implanted [67]. Our results showed how the lowest degradation values are obtained at 4 ± 0.5 °C and 70% HR for AL/HA-ink, and at 25 ± 0.5 °C with 35% HR for hMSC/AL/HA-bioink. Moreover, it could be observed how degradation of hMSC/AL/HA-bioink increased increased when the bioink was exposed to extreme conditions (40 °C and 70% HR). This fact was attributed to the stress that cells are subjected at non-physiological temperatures [72].

The swelling behavior is associated with the diffusion of nutrients and other molecules in the hydrogel, and it is controlled by the crosslinking process and the charge density. Swelling behavior is also influences to the final shape and the size of the printed 3D construct. The bioprinting resolution is another crucial parameter for bioprinting applications, which is affected by the swelling behaviour [67, 69]. The swelling values were higher at 35% RH for both AL/HA-ink and for hMSC/AL/HA-bioink. Besides, hMSC/AL/HA-bioink exhibited higher swelling ratio when compared to those of AL/HA-ink under the same conditions. Results showed high values of swelling ratio possibly due to hMSC/AL/HA-bioink presented hydrophilic nature [67, 73].

Porosity of materials influences the diffusion of nutrients, oxygen and metabolic products. Porosity can also facilitate the cellular migration, infiltration and directly affects the swelling capacity [66]. Our results showed that the porosity and pore size of the bioink was adequate for cell infiltration. The bioink designed in this study showed good ability to maintain high cell viability and proliferation over several days.

It is well-known that both, dynamic and static cell behavior, may be affected by the zeta potential [74]. Our results showed a negative zeta potential, surely due to the presence of hydroxyl and carboxyl groups on the surface of AL and HA [75]. A negative charge of the surface can interact with cell adhesion proteins, composed of hydrophobic amino acids or positively charged amino acids, such as arginine and lysine. Previous studies demonstrated that a negative surface may control both, cell-cell interactions and cell-material interactions [46]. On the other hand, high values of conductivity were obtained, which may contribute to induce the specific differentiation of hMSC, and enhance the cell-to-cell communication and cell adhesion [76].

During bioink development, it is important to determine its rheological behavior to ensure appropriate 3D-bioprinting properties. Developed formulations did not show thermo-sensitivity, and exhibited the same aspect of thick hydrogel at both low and high temperatures. Rheological studies also revealed a prevalence of the elastic behavior over the viscous behavior (G’ > G’’). AL/AH-bioink and hMSC/AL/HA-bioink exhibited differences with respect to their viscoelastic properties, being the values of G’, G’’ and 𝜂* smaller in the case of AL/HA-bioink. The cells addition provided a higher degree of structuration to the system. This fact is due to the spatial distribution of cells within a bioink. The specific volume occupied by cells, depending on their size and density, influences the viscoelastic properties and the cross-linking process. Cells can behave as a physical barrier between different regions of the bioink or restricting contact between reacting groups [60]. Diamantides et al. [77] demonstrated that the addition of cells alters the rheological properties of biomaterials. Our rotational testing results show that AL/HA-ink and hMSC/AL/HA-bioink displayed a shear thinning flow at 25 ± 0.5 °C and 37 ± 0.5 °C. The profiles of the viscosity curve for each bioink were quite similar at both temperatures. Additionally, viscosity results (at 100 s^-1^) and apparent thixotropy were higher for hMSC/AL/HA-bioink, confirming that the addition of cells promoted a higher degree of structuration to the system, and showing that the incorporation of these cells altered the rheological properties. This is because cells occupy a specific volume, which impacts on the cross-linking efficiency and viscoelastic properties [78]. Our bioink is primarily intended for application in volumes exceeding nano/micro scales. Consequently, rheological analysis was selected over the supplementary insights that may be gained from mechanical testing via nanoindentation [79, 80], since these are deemed negligible when compared to the comprehensive data offered by oscillatory and rotational testing. Therefore, the developed bioink revealed good rheological properties for its use in extrusion-based bioprinting methodology. This process requires the bioink to exhibit thixotropy properties, being appropriate to print highly viscous cell-laden bioinks [68].

Moreover, stress-stability studies did not reveal any signs of physical instability of the developed bioink. Therefore, the designed bioink is optimal to ensure cell retention.

The manufacture and storage of the hMSC/AL/HA-bioink should guarantee cell viability. Many studies analyze the effect of temperature due to its influence on the storage properties of different types of bioinks [65]. However, this effect of temperature over prolonged periods had not been previously analyzed. The results of our study indicate that the bioink loaded with hMSC achieved greater cell viability (85.1 ± 15.9%) overtime when the hMSC/AL/HA-bioink was stored at 4 ± 0.5 °C for 48 h without culture medium, showing a drastic decrease after 72 h. This is attributed to the metabolic activity of cells, which remain more stable at low temperatures during the first hours. Thus, these results determine that half-life of hMSC/AL/HA-bioink after its formulation was 48 h, keeping the cell viability values above 80%, being an acceptable viability for pre-bioprinting. According to the FDA and EMA requirements for advanced therapies medicinal products (ATMPs), the cell viability of an ATMP before to be administered should be above 70–80% [81].

We also examined the biocompatibility of hMSC/AL/HA-bioink to analyze its potential application for 3D bioprinting. Confocal images from LIVE/DEAD^®^ staining assay revealed high cell viability in hMSC/AL/HA-bioink, reaching values above 90% of living cells throughout the period of culture. These results are in accordance with previous studies about hydrogels for tissue engineering, in which a cell-friendly environment was provided by AL and HA as main components [14]. Cell proliferation inside hMSC/AL/HA-bioink indicated an increase in cell population over time, which was expected since HA has been widely demonstrated to stimulate proliferation in many other studies [82].

A bioink must be able to be extruded through the syringe of the bioprinter while maintaining its ability to retain the cells contained within it and their survival. Thus, the filament should be of similar width to the nozzle inlet diameter to facilitate the extrusion process [83]. A reduced nozzle size has the potential to create high shear stress and extensional strains on cells, thereby affecting the cell survivability negatively. Furthermore, the sizes of cells and other aggregates, besides their uneven distribution, may obstruct the nozzle or produce inconsistent filaments, thereby restricting the printing resolution [84]. The designed shape is also important to allow oxygen transfer, nutrient exchange, and the removal of cellular debris. Similarly, the maintenance of geometry is very important for load-bearing tissues [5, 85]. Therefore, to check if specifications remained consistent, the width of the extruded filament was measured after printing. Our results confirm the bioink formed a smooth and uniform filament of 0.4 mm width and no significant changes in the bioink’s shape were observed upon extrusion. Futhermore, fusion between lines of the same layer was not detected. Additionally, the bioprintability study of bioink showed high cell viability maintenance after being extruded by the bioprinter. These results highlight that the hMSC/AL/HA-bioink provides an optimal environment to promote cell proliferation and viability, and confirmed that the developed bioink fulfills all the requirements for tissue engineering.

## Conclusions

In this work, a semi-crosslinked hMSCs-loaded AL/HA-hydrogel was successfully developed to provide adequate physicochemical, mechanical, and biological characteristics for its use as bioink. From a physicochemical perspective, hMSC/AL/HA-bioink had an optimal porosity, swelling behaviour and appropriate surface properties and degradation rate. The hydrogel containing hMSCs also exhibited the required rheological behaviour for its bioprinting by extrusion. In addition, assayed stress-stability studies did not reveal any signs of physical instability, which is essential to ensure cell viability. The hMSC/AL/HA-bioink provided an appropriate environment for cell viability and growth. Based on our study, hMSC/AL/HA-bioink is regarded as a stable and suitable formulation for its use in 3D bioprinting and can be proposed as a potential candidate tools for tissue engineering and regenerative medicine.

## Supplementary information

Below is the link to the electronic supplementary material.

Supplementary Material 1 (DOCX 1470 KB)

## Data Availability

The data presented in this study are available on request from the corresponding authors.
