# Supplementary material for: Human mesenchymal stromal cells-laden crosslinked hyaluronic acid-alginate bioink for 3D bioprinting applications in tissue engineering
Source: Drug Deliv Transl Res. 2024 Apr 25;15(1):291–311. doi: 10.1007/s13346-024-01596-9 (PMC11614963; doi:10.1007/s13346-024-01596-9)
Supplement: Supplementary file 1 — Supplementary Material 1 (DOCX 1470 KB) [file 13346_2024_1596_MOESM1_ESM.docx]

**Supplementary Material**


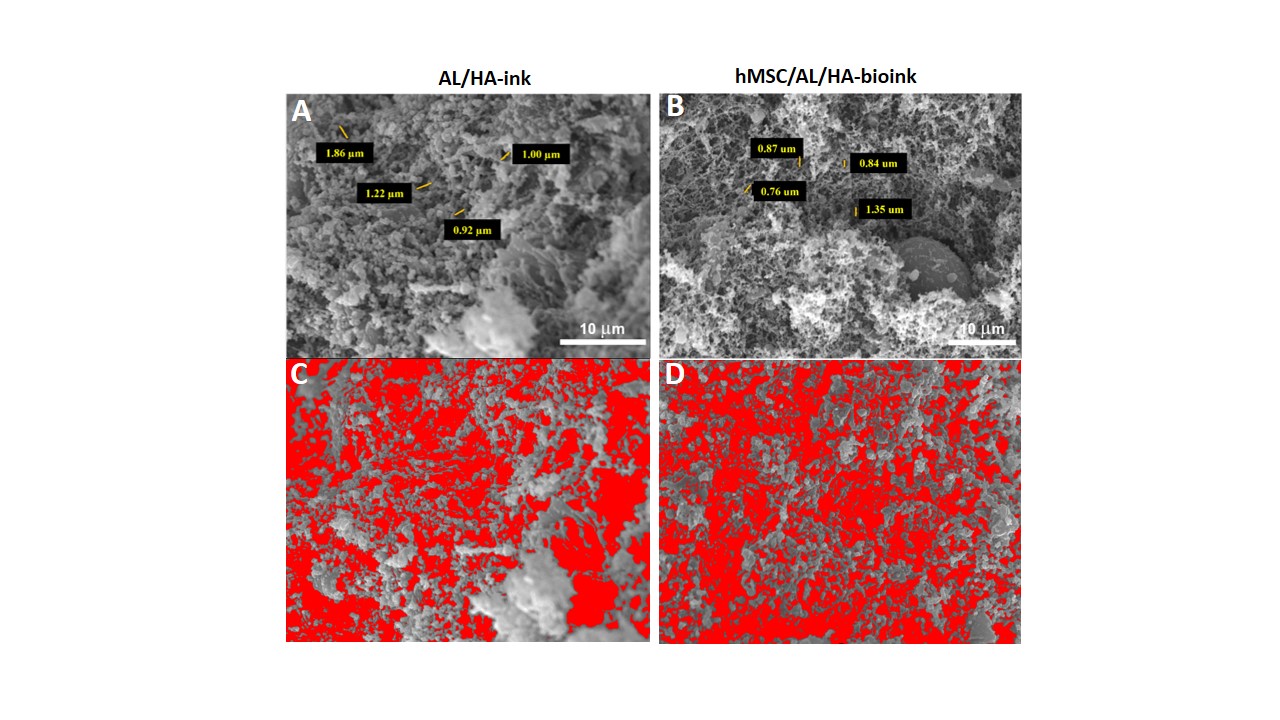


**Figure S1.** SEM images and average pore sizes of bioinks and SEM images of bioinks processed using ImageJ. (A) SEM image of AL/HA-ink, magnification: < 2,300×. (B) SEM image of hMSC/AL/HA-bioink, magnification: 2,200×. (C) SEM images of AL/HA-ink processed using ImageJ software. (D) SEM images of hMSC/AL/HA-bioink processed using ImageJ software.

| **A** |
| --- |
|  |
| **B** |
|  |

**Figure S2.** (A) Viscosity curve versus temperature (η = f (γ)) of AL/HA-ink and hMSC/AL/HA-bioink at 25 ± 0.5 °C. (B) Viscosity curve versus temperature (η = f ɣ̇) of AL/HA-ink and hMSC/AL/HA-bioink at 37± 0.5 °C.

**

**Figure S3.** Images of healing-cooling cycle, freeze-thaw cycle and centrifugation cycle of the AL/HA-ink.


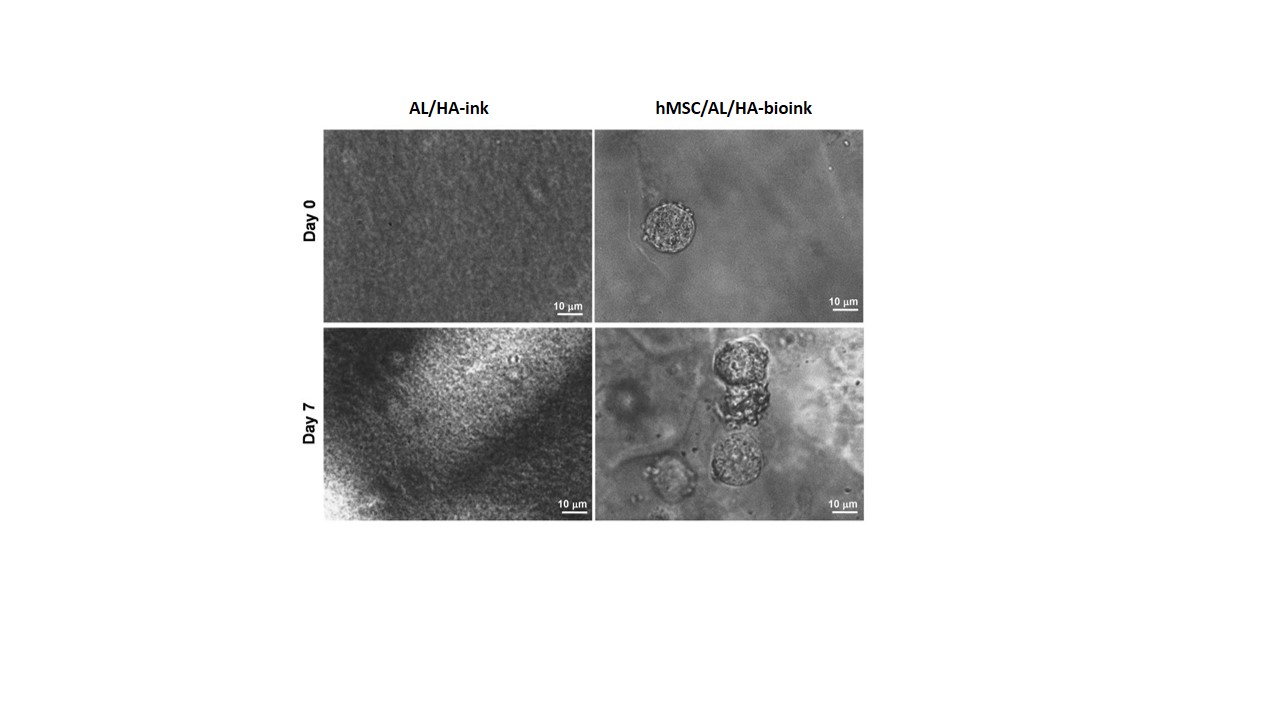


**Figure S4.** Optical morphology of AL/HA-ink and hMSC/AL/HA-bioink at different time points.

**Table S1.** Results of the osmolality of bioinks.

| **Formulation** | **Osmolality (Osm/kg)** |
| --- | --- |
|  |  |
| AL/HA-ink | 0.110 ± 0.003 |
| hMSC/AL/HA-ink | 0.116 ± 0.003 |

**Table S2.** Results of the rheological rotational testing of bioinks at different temperatures of measurement.

| **Formulation** | **Temperature  (º C)** | **Mathematical Model Fitting** | | **Rheological Behavior** | **Viscosity at 100 s^-1^ (Pa·s)** | | **Hysteresis loop area (Pa/s)** |
| --- | --- | --- | --- | --- | --- | --- | --- |
|  |  | **Ramp-Up**  **Strech** | **Ramp-Down Strech** |  | |  |  |
| AL/HA-ink | 25 | Herschel-Bulkley  r = 0.9993 | Cross  r = 0.9999 | Shear thinning | | 0.255 ± 3.29e^-3^ | 371.2 |
|  | 37 | Herschel-Bulkley  r = 0.9985 | Cross  r = 0.9999 | Shear thinning | | 0.225 ± 2.87e^-3^ | 325.3 |
| hMSC/AL/HA-bioink | 25 | Herschel-Bulkley  r = 0.9985 | Cross  r = 0.9999 | Shear thinning | | 3.761 ± 3.68e^-2^ | 9075 |
|  | 37 | Herschel-Bulkley  r = 0.9975 | Cross  r = 1 | Shear thinning | | 3.446 ± 1.74e^-2^ | 11270 |
